# The functional characterization of phosphorylation of tristetraprolin at C-terminal NOT1-binding domain

**DOI:** 10.1186/s12950-021-00288-2

**Published:** 2021-06-05

**Authors:** Hsin-Hui Hsieh, Yen-An Chen, Yao-Jen Chang, Hsin-Hui Wang, Ya-Han Yu, Sheng-Wei Lin, Yin-Jung Huang, Steven Lin, Ching-Jin Chang

**Affiliations:** 1 Graduate Institute of Biochemical Sciences, College of Life Science, National Taiwan University, No. 1 Sec 4 Roosevelt Rd, Taipei, 106 Taiwan; 2 Institute of Biological Chemistry, Academia Sinica, Taipei, Taiwan; 3 Department of Pediatrics, Division of Pediatric Immunology and Nephrology, Taipei Veterans General Hospital, Taipei, Taiwan; 4 Department of Pediatrics, Faculty of Medicine, School of Medicine, National Yang Ming Chiao Tung University, Taipei, Taiwan; 5 Institute of Emergency and Critical Care Medicine, School of Medicine, National Yang Ming Chiao Tung University, Taipei, Taiwan

**Keywords:** Tristetraprolin, Phosphorylation, CNOT1, deadenylase

## Abstract

**Background:**

Tristetraprolin (TTP) family proteins contain conserved tandem CCCH zinc-finger binding to AU-rich elements and C-terminal NOT1-binding domain. TTP is phosphorylated extensively in cells, and its mRNA destabilization activity is regulated by protein phosphorylation.

**Methods:**

We generated an antibody against phospho-Serine316 located at the C-terminal NOT1-binding site and examined TTP phosphorylation in LPS-stimulated RAW264.7 cells. Knockout of TTP was created in RAW264.7 cells using CRISPR/Cas9 gene editing to explore TTP functions.

**Results:**

We demonstrated that Ser316 was phosphorylated by p90 ribosomal S6 kinase 1 (RSK1) and p38-activated protein kinase (MK2) and dephosphorylated by Protein Phosphatase 2A (PP2A). A phosphorylation-mimic mutant of S316D resulted in dissociation with the CCR4-NOT deadenylase complex through weakening interaction with CNOT1. Furthermore, Ser316 and serines 52 and 178 were independently contributed to the CCR4-NOT complex recruitment in the immunoprecipitation assay using phosphor-mimic mutants. In RAW264.7 macrophages, TTP was induced, and Ser316 was phosphorylated through RSK1 and MK2 by LPS stimulation. Knockout of TTP resulted in *TNFα* mRNA increased due to mRNA stabilization. Overexpression of non-phosphorylated S316A TTP mutant can restore TTP activity and lead to *TNFα* mRNA decreased. GST pull-down and RNA pull-down analyses demonstrated that endogenous TTP with Ser316 phosphorylation decreased the interaction with CNOT1.

**Conclusions:**

Our results suggest that the TTP-mediated mRNA stability is modulated by Ser316 phosphorylation via regulating the TTP interaction with the CCR4-NOT deadenylase complex.

**Supplementary Information:**

The online version contains supplementary material available at 10.1186/s12950-021-00288-2.

## Background

Transcriptome dynamics are governed by RNA synthesis and degradation. Regulation of mRNA stability plays a central role in controlling gene expression in the vast majority of eukaryotic cells [1–4]. The mRNA stability is modulated by *cis*-acting elements in the 3′ untranslated region **(**UTR) as well as *trans*-acting factors [5, 6]. The AU-rich element (ARE) is the best-studied *cis*-acting element in short-lived mRNA [7]. Several ARE-binding proteins have been shown to regulate mRNA turnover/decay [8], which is initiated by deadenylation via deadenylases including poly(A)-specific ribonuclease (PARN) and the polyA nuclease 2 (PAN2)-PAN3 and carbon catabolite repression (CCR4)-negative on TATA-less (NOT) complexes [9]. CCR4-NOT is a highly conserved multisubunits molecular machine with an approximate molecular mass of 1 MDa that contains two deadenylases, CCR4 (also named CNOT6) and CAF1 (also named CNOT7) [10, 11]. The largest subunit of CCR4-NOT, namely CCR4-NOT complex subunit 1 (CNOT1), serves as a hub of protein-protein interactions [11]. Specific mRNAs can be targeted by RNA-binding proteins, such as ARE-containing mRNAs can be recognized by tristetraprolin (TTP), to recruit deadenylase complexes for mRNA degradation [12, 13].

TTP is an extensively studied ARE-binding protein. There are four TTP members in rodents, including TTP, Zfp36l1, Zfp36l2, and Zfp36l3, and they all contain conserved tandem CCCH zinc finger RNA–binding domains and a conserved C-terminal NOT1-binding domain [14]. TTP contains intrinsically unstructured regions outside these two conserved regions [15–17]. The metazoa type of NOT1-binding domain is missing in most fungi; for examples, the TTP homologs CTH1 and CTH2 in *Saccharomyces cerevisiae*, and Zfs1p in *S. pombe*, are no containing NOT1-binding domain [14]. TTP was induced by lipopolysaccharide (LPS) and served as an anti-inflammatory factor to inhibit cytokine expression such as TNF-α [18]. We demonstrated that Zfp36l1 and Zfp36l2 proteins were maintained at a constant level and were phosphorylated under LPS stimulation [19]. Zfp36l3 expression is limited to the placenta and yolk sac, and is important for overall fecundity [20]. TTP family proteins are serine/threonine-rich, and they appear as multiple bands in SDS-PAGE, indicating that they are highly phosphorylated [21, 22]. TTP can be phosphorylated by mitogen-activated protein kinase (MAPK) p38-activated protein kinase 2 (MK2) at serines 52 and 178 in mouse macrophages to allow binding of 14–3-3 adaptor proteins, which inhibits the mRNA destabilizing activity of TTP [23, 24]. In contrast, PP2A can compete with 14–3-3 proteins to dephosphorylate TTP at Ser178 and thereby activate decay of cellular mRNAs [25, 26]. The substitution of these two serines (Ser52 and Ser178) to non-phosphorylated alanines in the endogenous murine locus encoding TTP gave rise to a strong and dominant anti-inflammatory phenotype [27]. One molecular mechanism for TTP-mediated mRNA decay is the recruitment of the CCR4-NOT deadenylase complex through direct interaction with CNOT1, resulting in the decay of ARE-containing mRNAs [28, 29]. TTP phosphorylated by MK2 inhibits the deadenylase recruitment due to association with 14–3-3 [30, 31]. In addition to the p38-MK2 pathway, ERK signaling has been reported to regulate protein stability, subcellular localization, and function of TTP [32, 33].

Previously, Ser316 phosphorylation was identified by mass spectrometry analysis in both in vitro MK2 phosphorylation and LPS-stimulated RAW264.7 macrophages [27]. Ser316 is located in the C-terminal conserved region of TTP family proteins, and this region is critical for CCR4-NOT complex recruitment [14, 28, 34]. In a SILAC (stable isotope labeling by amino acids) analysis, Ser316 phosphorylation might be an additional residue responding to MK2/3 in addition to the Ser52/Ser178 [35]. In this study, we generated a specific antibody against phospho- Ser316 of TTP to examine the Ser316 phosphorylation under LPS stimulation in mouse RAW264.7 macrophages and find out the possible kinases and phosphatases. We also created phosphomimetic mutant (S316D) and non-phosphorylated mutant (S316A) to demonstrate that the Ser316 phosphorylation would inhibit CCR4-NOT complex recruitment. The TTP knockout RAW264.7 cells were created by CRISPR/Cas9 gene editing, after transfected with TTP S316A mutant would lead to downregulation of TTP –targeted mRNA. The results confirmed that Ser316 phosphorylation of TTP plays an important function in TTP-mediated mRNA destabilization in LPS-stimulated RAW264.7 cells.

## Materials and methods

### Cell culture

Human embryonic kidney (HEK) 293T cells (CRL-3216) and mouse NIH3T3 cells (CRL-1658) were purchased from American Type Culture Collection (ATCC) and were cultured in DMEM containing 3.7 g/l sodium bicarbonate and supplemented with 10% FBS (Gibco), 100 U/ml penicillin, and 100 mg/ml streptomycin (Gibco) in a 5% CO_2_ humidified atmosphere (37 °C). Mouse BALB/c macrophage RAW264.7 cells (TIB-71) were purchased from ATCC and were cultured in Roswell Park Memorial Institute (RPMI) 1640 supplemented with 10% FBS (Gibco), 100 U/ml penicillin, and 100 mg/ml streptomycin (Gibco) in a 5% CO2 humidified atmosphere (37 °C). The RAW264.7 cells were treated with 100 ng/ml of LPS from *E. coli* O111:B4 (Sigma-Aldrich) for different time intervals, combined with the 2 or 5 μM of MK2 inhibitor PF3644022 (Sigma-Aldrich) and 25 or 50 μM of RSK1 inhibitor BI-D1870 (Abcam).

### Plasmid constructs

The plasmids for TTP, Zfp36l1, Zfp36l2 and 14–3-3ζ expression were constructed as described [19, 21, 22, 36]. The S316 and S318, S52, and S178 mutants in TTP were created by PCR (for S316 and S318) or Q5 site-directed mutagenesis kit (New England Biolabs) (for S52 and S178) using the primers indicated in Table S1. The PCR products were ligated to pCMV-Tag2 (Stratagene) and pEGFP-C2 (Clontech) for mammalian cell expression. Mouse Cnot1@800–1310 was PCR amplified from a full- length Cnot1 cDNA template (OriGene) with the primers: 5′-CAGGCTCAGGCCCAGGTT-3′ and 5′-TTATTAGGCCTGAGCCAGTGCAATAC-3′. The PCR products were cloned into pGEX4T-1 to express glutathione S-transferase (GST)-fused proteins in bacteria.

### RNA isolation, reverse transcription, and quantitative PCR

Total RNA was extracted from cell cultures using TRIzol reagent (Invitrogen). For mRNA stability analysis, the cells were treated with 10 μg/ml actinomycin D (transcription inhibitor) for various times to inhibit new transcription. After DNase I digestion, 2 μg total RNA was reverse-transcribed to produce cDNA using M-MLV reverse transcriptase and oligo dT primer (Promega). Real-time PCR was performed with the 7300 Real-Time PCR System (Applied Biosystems) in a total volume of 20 μl. Expression of genes encoding TTP, TNFα and actin was assessed using SYBR Green PCR Master Mix (Applied Biosystems) with 50 ng of cDNA and 160 nM of each primer: 5′-GGATCTCTCTGCCATCTACGA-3′ and 5′-CAGTCAGGCGAGAGGTGAC-3′ for TTP; 5′-GACCCTCACACTCAGATCATCTTCT-3′ and 5′-CCTCCACTTGGTGGTTTGCT-3′ for TNFα; 5′-TCCTTCCTGGGCATGGAGTC-3′ and 5′-ACTCATCATACTCCTGCTTG-3′ for β-actin. The PCR amplification conditions were 40 cycles of 95 °C for 15 s and 60 °C for 1 min. Real-time PCR data were analyzed using the 2^–∆∆CT^ relative quantitation method.

### Cell extracts preparation, co-immunoprecipitation (IP), and western blotting

HEK293T cells were transfected with the plasmids using Turbofect reagent (Thermo). Cells harvested 24 h after transfection were lysed with NET buffer (50 mM Tris, pH 7.5, 150 mM NaCl, 1 mM EDTA, 0.1% (v/v) Triton X-100) containing a protease inhibitor cocktail (Sigma-Aldrich) and phosphatase inhibitors (10 mM β-glycerol phosphate, 0.1 mM Na_2_MoO_4_, 0.1 mM Na_3_VO_4_, pH 10.0, 10 mM NaF) and centrifuged at 15,000×*g* for 10 min. The supernatants were immunoprecipitated using anti-Flag M2 agarose (Sigma-Aldrich) at 4 °C for 2 h. After the IP mixture was washed three times with NET buffer, bound proteins were eluted by boiling in SDS-PAGE sample buffer. The proteins were separated by SDS-PAGE (10% polyacrylamide) and transferred to a polyvinylidene difluoride membrane (Millipore), and western blotting was performed using anti-Flag (1:2000)(Sigma-Aldrich), anti-HA (1:2000) (Bethyl Laboratories), anti-TTP and anti-p-S316 (1:1000)  (produced in our lab), anti-α-tubulin (1:1000), anti-β-actin (1:1000), anti-phospho-ERK1/2 (1:1000), anti-p38 (1:1000), anti-phosph-p38 (1: 1000), anti-MK2 (1:1000), anti-phospho-MK2 (1:200), anti-phospho-RSK1(1:200), anti-CNOT3 (1:1000) and anti-CNOT6 (1:1000) (all from Cell Signaling Technology), anti-GAPDH (1:5000), anti-CNOT1 (1:1000), and anti-CNOT7 (1:500) (all from Proteintech Group), anti-ERK1/2 (1:2000) and anti-RSK1(1:2000) (both from Santa Cruz Biotechnology), and anti-DDX6 (1:1000)(Abcam). All experiments were carried out at least three times, and represented results were displayed.

### Generation of rabbit anti-phospho-S316 of TTP

A peptide containing the sequence surrounding phospho-S316 of TTP (RLPIFNRIpSVSE) was synthesized and purified by Kelowna International Scientific Inc. (Taiwan). A specific rabbit antiserum was produced by LTK BioLaboratories (Taiwan). The antiserum was affinity purified using the immunizing peptide (LTK BioLaboratories).

### In vitro kinase assay

Each reaction mixture contained 2 μg of recombinant GST-tagged TTP (wild type or mutants) served as substrates, 3 μl of 10X reaction buffer (New England Biolabs), 30 μM of ATP, and kinases including ERK2 (New England Biolabs), p38 alpha (SignalChem), RSK1 (SignalChem), and MK2 (SignalChem) in a final volume of 30 μl. The kinases can be from cell extracts. 300 μg of LPS-treated RAW264.7 whole cell extracts were incubated with GSH-Sepharose bound 2 μg of GST-TTP in the buffer containing 20 mM HEPES, pH 7.7, 75 mM NaCl, 0.5 mM MgCl_2_, 0.1 mM EDTA, 0.05% Triton X-100, 0.5 mM DTT, 20 mM β-glycerolphosphate, 0.1 mM Na_3_VO_4_, 1 μg/ml leupeptin, 1 μg/ml pepstatin A, 100 μg/ml PMSF. The mixture was rotated at 4 °C for 3 h and pelleted by centrifugation at 10,000×g for 20 s. After 4 × 1-ml washes in HEPES binding buffer (20 mM HEPES, pH 7.7, 50 mM NaCl, 2.5 mM MgCl_2_, 0.1 mM EDTA, 0.05% Triton X-100), the beads were resuspended in 30 μl for kinase assay. The reaction mixtures were incubated at 30 °C for 30 min and stopped by adding one volume of protein sample buffer. Samples were subjected to SDS-PAGE for western blotting with anti-phospho-S316 and ponceau S staining.

### GST pull-down assays

Glutathione-Sepharose 4B (~ 8 μl, GE Healthcare Life Sciences) were incubated with 2 μg of bacterially expressed GST, or GST-Cnot1@800–1015 or GST-14-3-3 in phosphate-buffered saline containing 1% (v/v) Triton X-100 on a rotary shaker for 20 min at room temperature. After washing three times with the same buffer, the Sepharose wa**s** combined with lysates (300 μg protein) of RAW264.7 cells that had undergone various treatments in a final volume of 200 μl of buffer containing 20 mM HEPES, pH 7.9, 100 mM NaCl, 2.5 mM MgCl_2_, 0.1 mM EDTA, 0.05% (v/v) NP-40, 1% (v/v) Triton X-100, 1 mM DTT, and 1 mM PMSF. The mixtures were incubated at 4 °C for 2 h on a rotary shaker, and then the Sepharose was washed four times with the same buffer lacking DTT and PMSF but containing 0.2 M NaCl and once with 50 mM Tris, pH 6.8. Bound proteins were eluted by boiling in SDS-PAGE sample buffer and analyzed by western blotting.

### RNA pull-down assays

Cytoplasmic extracts from LPS-stimulated RAW264.7 cells were prepared by hypotonic buffer (10 mM HEPES, pH 7.5, 10 mM potassium acetate, 1.5 mM magnesium acetate, 2.5 mM DTT, 0.05% NP-40, and protease inhibitor cocktails). Potassium acetate was adjusted to 90 mM, and 0.1 U·μL^− 1^ RNasin (Promega) and 20 μg·μL^− 1^ yeast tRNA was added to each lysate. To prevent non-specific binding, heparin-agarose (Sigma-Aldrich) was incubated with each lysate for 15 min at 4 °C and then centrifuged for 1 min at 8000 rpm, 4 °C. Each supernatant was further cleaned with streptavidin-Sepharose (8 μL**;** Invitrogen) for 1 h at 4 °C and then centrifuged for 1 min at 8000 rpm, 4 °C. The biotin-labeled *TNFα* ARE was added as described [19]. The pulled-down RNA-protein complexes were washed four times with binding buffer (hypotonic buffer containing 90 mM potassium acetate) and separated by SDS-PAGE (10% acrylamide) for western blotting analysis.

### siRNA-mediated knockdown, transfection and immunoprecipitation assay

HEK293T cells (1 × 10^5^) were seeded in each well of a 12-well plastic culture plate. For gene knockdown, the cells were transfected with 5 nM of a small interfering RNA (siRNA) for CNOT1, CNOT6, CNOT7, RSK1, or MK2 (Invitrogen) using Lipofectamine 3000 (Invitrogen). After 24 h, the cells were transferred into a fresh medium and transfected with 1 μg of Flag-TTP wild-type, −TTP S52,178A or –TTP S316A expression plasmids using Turbofect reagent (Thermo). After another 24 h, cells were harvested, and whole cell extracts were isolated for western blotting or immunoprecipitation.

### CRISPR/Cas9-mediated gene editing

CRISPR-based sgRNAs were designed on Benchling (https://benchling.com) and CHOPCHOP (https://chopchop.cbu.uib.no/) to search the specific target-sequences of Cas9 RNP complexes on mouse *Ttp* gene. Based on the description of the target score [37] (https://crispr.mit.edu/about), we designed four sgRNAs, which contain a relatively higher on-target with a lower off-target score (Table S2). Each DNA template of TTP sgRNA encoding for a T7 promoter, a 20 nt target sequence, and a published sgRNA scaffold [38] were assembled by overlapping PCR. Each PCR reactions contain 20 nM premix of TTP sgRNAs and bottom scaffold (Table S2), 1 μM premix of T7 oligo primer and sgRNA-reverse, 200 μM dNTP, and Q5 polymerase (NEB) according to the manufacturer’s protocol. The thermocycler setting consisted of 30 cycles of 95 °C for 10 s, 59 °C for 10 s and 72 °C for 10 s [39].

The assembled PCR products were extracted once with phenol:chloroform:isoamyl alcohol and then once with chloroform, before isopropanol precipitation overnight at − 20 °C. The DNA pellet was washed three times with 70% ethanol and dissolved in DEPC-treated water. The T7 in vitro transcription reaction consisted of 30 mM Tris–HCl (pH 7.9), 20 mM MgCl_2_, 0.01% Triton X-100, 2 mM spermidine, 10 mM DTT, 5 mM of ribonucleotide triphosphate, 100 μg/ml T7 polymerase and 1 μM DNA templates. The reaction was incubated at 37 °C for 4 h, and RNase-free DNase was added to digest the DNA template 37 °C for 1 h. The reaction was stopped by adding 2xSTOP solution (95% deionized formamide, 0.05% bromophenol blue and 20 mM EDTA) at 60 °C for 5 min. The RNA was purified by electrophoresis in 10% polyacrylamide gel containing 6 M urea. The RNA band was excised from the gel, ground up in a 15-ml tube, and eluted with 5 vol. of 300 mM sodium acetate (pH 5.0) overnight at 4 °C. One equivalent of isopropanol was added to precipitate the RNA at − 20 °C. The RNA pellet was centrifuged and washed three times with 70% ethanol and dried by vacuum. To refold the sgRNA, the RNA pellet was first dissolved in 20 mM HEPES (pH 7.5), 150 mM KCl, 10% glycerol, and 1 mM TCEP. The sgRNA was heated to 70 °C for 5 min and cooled to room temperature. MgCl_2_ was added to a final concentration of 1 mM. The sgRNA was again heated to 50 °C for 5 min, cooled to room temperature and kept on ice. The sgRNA concentration was determined by OD_260nm_ and adjusted to 100 μM using 20 mM HEPES (pH 7.5), 150 mM KCl, 10% glycerol, 1 mM TCEP, and 1 mM MgCl_2_. The sgRNA was store at − 80 °C [39].

Transfection of RAW264.7 cells was performed according to the instructions of Lipofectamine™ CRISPRMAX™ Cas9 Transfection Reagent (Invitrogen). The Cas9 RNP were prepared by incubating the purified Cas9 protein with sgRNA at 1:4 molar ratios. After transfection, the cells were incubated at 37 °C for 48 h, and followed by single-cell sorting using BD, FACSJazz automated cell sorter to perform the single isolation (TechComm at NTU).

### Genomic DNA isolation and PCR analysis

Genomic DNA was extracted by QuickExtract™ DNA Extraction Solution (Epicentre). Cells were mixed by 200 μl of QuickExtract Solution and vortex for 15 s. The tubes were transferred to 65 °C and incubate for 6 min. After 15 s vortex, samples were incubated at 98 °C for 2 min. Store the DNA at − 20 °C or − 80 °C for long-term storage according to the manufacturer’s protocol. Genomic DNA was PCR amplified using primer-1 and primer-4 (Table S2). Theoretically, sgRNA mTTP KO-1 and sgRNA mTTP KO-4 will generate double-strand DNA breaks on each target site, and cause a 793 nt deletion between exon 1 and exon 2 on the *Ttp* gene in RAW264.7 cells. Therefore, the size of PCR product generated by primer-1 and primer-4 will be reduced from 2001 nt to 1208 nt. Besides, primer-2 and primer-3 locate on the predicted cutting fragment. If the TTP knock-out happened, it would not have PCR products between primer-1 and primer-2 or primer-3 and primer-4 theoretically (Table S2).

### Indirect immunofluorescence staining

NIH3T3 cells were typically seeded at 50% on sterile glass coverslips before 16–18 h. After transfected with GFP-fused TTP expression vector using Lipofectamine 2000 (Thermo) for 24 h and treated with 20 ng/ml of leptomycin B (LMB) for 6 h, cells were washed briefly with PBS and then fixed in 4% paraformaldehyde/PBS for 30 min at room temperature. After gently washed twice with PBS (5 min for each), cells were permeabilized with 0.1% Triton X-100/PBS for 10 min at room temperature. Following another two times washed with PBS (5 min for each), blocked the cells for 30 min with PBS containing 1% BSA. Then the cells were incubated with appropriately diluted DDX6 antibody overnight at 4 °C, washed with PBS three times (10 min for each), further incubated with secondary antibody (Alexa Fluor 594 goat anti-rabbit Ig, molecular probe) and DAPI (Sigma-Aldrich) for 1.5 h in the dark, and washed with PBS three times (10 min for each) in the dark. Mount in mounting fluid and store at − 20 °C until detected by Leica SP5 confocal Microscopy system.

### Statistical analysis

All data are presented as the mean ± SD of at least three independent experiments. Statistical significance (*P < 0.05, **P < 0.01 or ***P < 0.001) was determined by one-tailed Student’s *t*-test.

## Results

### Ser316 of TTP is phosphorylated by RSK1 and MK2

**Fig. 1:**
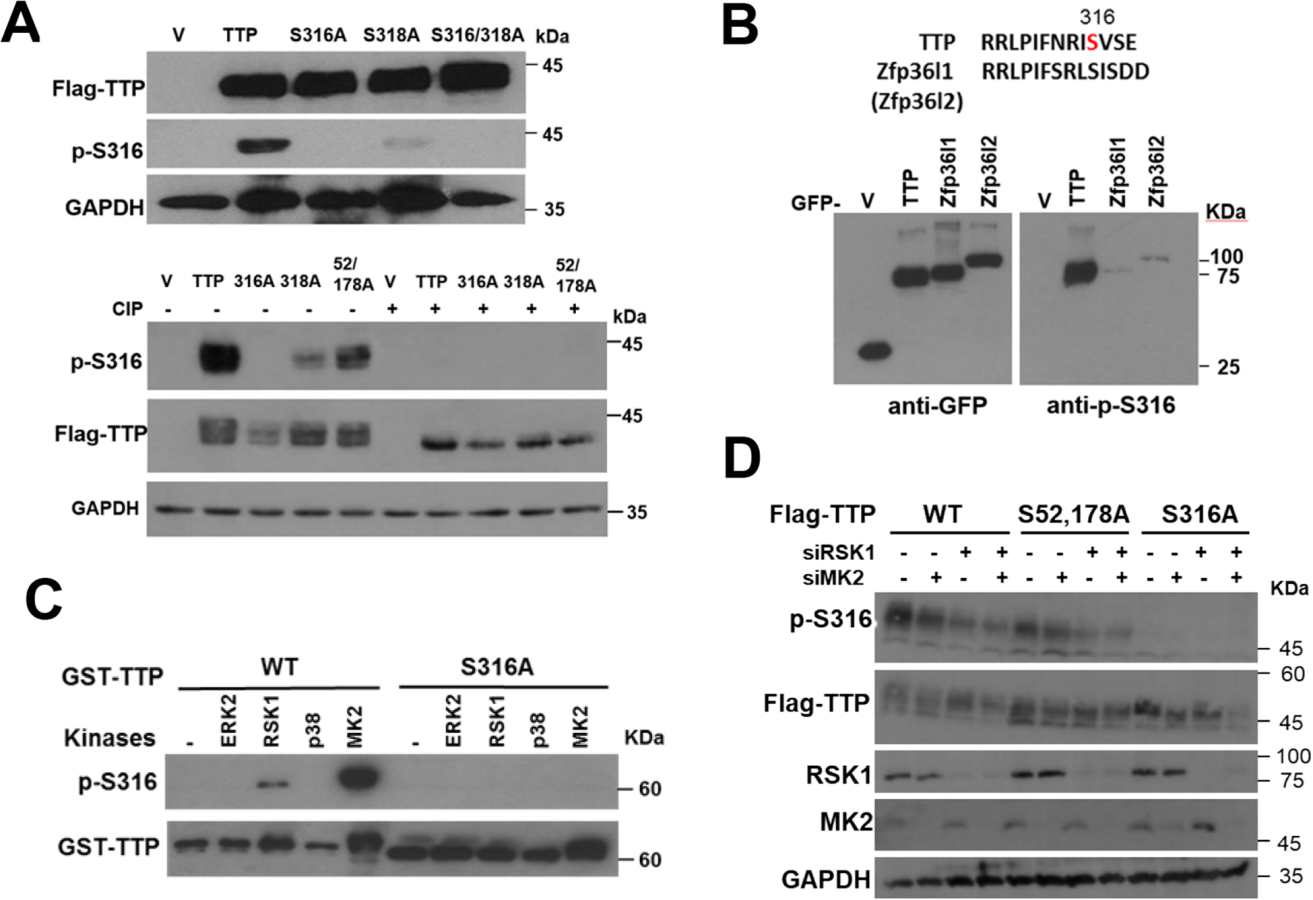
Generation of a specific antibody against phospho-Ser316 (p-S316) of TTP. **A** The antibody specifically recognizes p-S316. HEK293T cells were overexpressed with wild-type, or S316A, or S318A, or S316,318A, or S52,178A of Flag-TTP. Cell extracts were isolated and with or without alkaline phosphatase treatment for western blotting analysis by using anti-TTP, anti-phospho-S316, and anti-Flag. **B** The antibody is specific for TTP not for Zfp36l1 and Zfp36l2. The amino acid sequences of NOT1-binding domain in TTP and Zfp36l1 (or Zfp36l2) were shown. HEK293T cells were transfected with pEGFP vector (V) or pEGFP- TTP family, TTP, Zfp36l1, or Zfp36l2, cell extracts were western blotting with anti-p-S316 and anti-GFP. **C** In vitro kinase assay. Recombinant GST-TTP(WT) or GST-TTP(S316A) was incubated with recombinant active kinases as indicated and then subjected to SDS-PAGE for western blotting by using anti-p-S316 and anti-TTP. **D** Knockdown of MK2 or RSK1 decreases Ser316 phosphorylation. HEK293T cells were transfected with siRNA of MK2 or RSK1 for 24 h and then overexpressed with Flag-TTP wild-type, S316A or S52, 178A. Cell extracts were harvested for western blotting analysis by anti-p-S316 and anti-Flag. All experiments were reproduced at least three times and the representative results were displayed

To detect Ser316 phosphorylation in physiological conditions, a specific antibody against phospho-Ser316 (p-S316) was generated, and its specificity was established using wild-type or mutant TTP (Fig. 1 A). The signals were disappeared after treated with calf-intestinal alkaline phosphatase, suggesting this antibody was phospho-specific. Although TTP family proteins such as Zfp36l1 and Zfp36l2 have conserved NOT1-binding domain, this anti-p-S316 specifically recognized TTP but not Zfp36l1 and Zfp36l2 (Fig. 1 B). Enhancing either ERK or p38 signaling pathways by overexpression of MKK1 or MKK3, respectively, increased Ser316 phosphorylation (Fig. S1A). The sequence of Ser316 is similar to the consensus sequence recognized by the protein kinase A, G, and C family [40]. The ERK downstream kinase RSK and p38 downstream kinase MK2 belong to this family. In vitro kinase analysis combined with western blotting showed that recombinant RSK1 and MK2 but not ERK2 and p38 could phosphorylate GST-TTP at Ser316 (Fig. 1 C). Knockdown of MK2 and RSK1 using siRNA in 293 T cells decreased Ser316 phosphorylation in overexpression of wild-type and S52,178A mutant (Fig. 1 D). Serines 52 and 178 of TTP have known to be phosphorylated by MK2 [24], and our result showed that Ser316 is another MK2-phosphorylated residue. The Ser316 phosphorylation was decreased under overexpression of wild-type PP2A (Fig. S1B), indicating PP2A dephosphorylates Ser316 phosphorylation. Taken together, we generate a specific antibody against Ser316 of TTP and demonstrate that Ser316 can be phosphorylated by MK2 and RSK1 and dephosphorylated by PP2A.

### Ser316 phosphorylation prevents TTP interaction with CCR4-NOT deadenylase complex

**Fig. 2:**
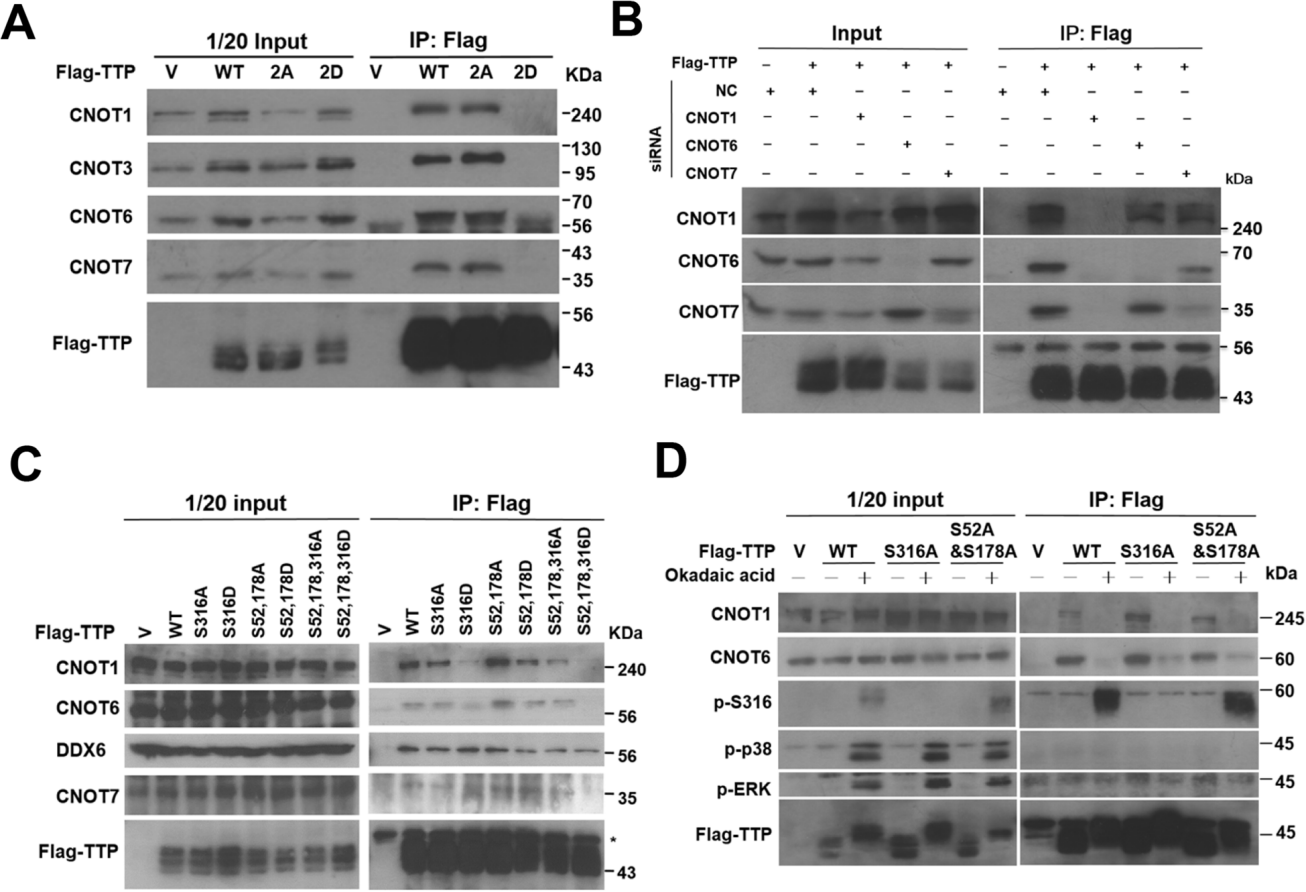
Ser316 phosphorylation decreases interaction with the CNOT1 complex and suppresses TTP activity. **A** Immunoprecipitation analysis. HEK293T cells were transfected with indicated Flag-TTP constructs and whole cell extracts were immunoprecipitated by anti-Flag. The precipitated protein complexes were analyzed by western blotting with antibodies against the CCR4-NOT complex as indicated. The asterisk indicates non-specific signals. **B** TTP directly interacts with CNOT1. HEK293T cells were transfected with siRNA specific for CNOT1, CNOT6, or CNOT7 or a negative-control siRNA (NC). After 24 h, cells were transfected again with the Flag-TTP plasmid. IP was performed with anti-Flag agarose and precipitated protein complexes were detected with antibodies against CNOT1, CNOT6, CNOT7, and Flag. **C** Immunoprecipitation analysis. HEK293T cells were transfected with indicated Flag-TTP constructs and whole cell extracts were immunoprecipitated by anti-Flag. The precipitated protein complexes were analyzed by western blotting with indicated antibodies. The asterisk indicates antibody heavy chain signals. **D** HEK293T cells were transfected with WT, S316A, or S52,178A Flag-TTP and treated with or without 1 μM of okadaic acid for 1 h. Whole cell extracts were immunoprecipitated with anti-Flag and western blotting with indicated antibodies. The asterisk indicates antibody heavy chain signals

The NOT1-binding domain contains two serines, Ser316 and Ser318, which might be phosphorylated to affect CNOT1-binding. The phosphomimetic mutant (S316,318D; S2D) and non-phosphorylated mutant (S316,318A; S2A) were created for co-immunoprecipitation analysis. Compared to wild-type and non-phosphorylated S316,318A mutant, the S316,318D phosphomimetic mutant weakened associations with CNOT1, CNOT3, CNOT6 and CNOT7 (Fig. 2 A). Moreover, the single mutant of S316D but not S318D decreased interaction with CNOT1 (Fig. S2A). Also, it decreased the suppressive activity of TTP on ARE-mediated luciferase analysis (Fig. S2B), indicating Ser316 phosphorylation plays a function in the CCR4-NOT complex-mediated RNA decay. When CNOT1 was knocked down, TTP could not associate with CNOT6 and CNOT7 in the co-IP experiments (Fig. 2 B). In CNOT7-knockdown cells, the precipitated CNOT6 was less abundant than in control cells (Fig. 2 B). These results suggest that TTP interacts with CNOT1 directly to recruit the CNOT6 and CNOT7 deadenylases, and the recruitment of CNOT6 is dependent on CNOT7. The knockdown of CNOT1 showed a stronger effect in perturbing the TTP function than the knockdown of CNOT6 or CNOT7 (Fig. S2C). It is known that MK2 phosphorylates mouse TTP majorly at Ser52 and Ser178 and creates a functional 14–3-3 binding site and further prevents deadenylase complex recruitment [23, 24, 31], and Ser316 is a minor MK2-phosphorylated residue [23]. We want to compare the CCR4-NOT complex recruitment activity between the mutants of both residues of serines 52 and 178 and Ser316. As shown in Fig. 2 C, phosphomimetic mutants of S316D and S52,178D exhibited poor association with CNOT1 and three residues mutant of S52,178,316D totally lost the interaction with the CCR4-NOT complex. The precipitated CNOT6 and CNOT7 deadenylases were also decreased in the phosphomimetic mutants (Fig. 2 C and Fig. S2D). When treated with PP2A inhibitor okadaic acid to activate p38 and ERKs signaling pathways, TTP was highly mobility-shifted as hyper-phosphorylated including phosphorylated at Ser316 (Fig. 2 D). Under this condition, not only wild-type TTP decreased interaction with CCR4-NOT complex but also mutants of S316A and S52, 178A did. These results imply that TTP phosphorylation at either serines 52 /178 or Ser316 independently decreases CCR4-NOT deadenylase complex recruitment.

### Ser316 of TTP is phosphorylated in LPS-stimulated RAW264.7

**Fig. 3:**
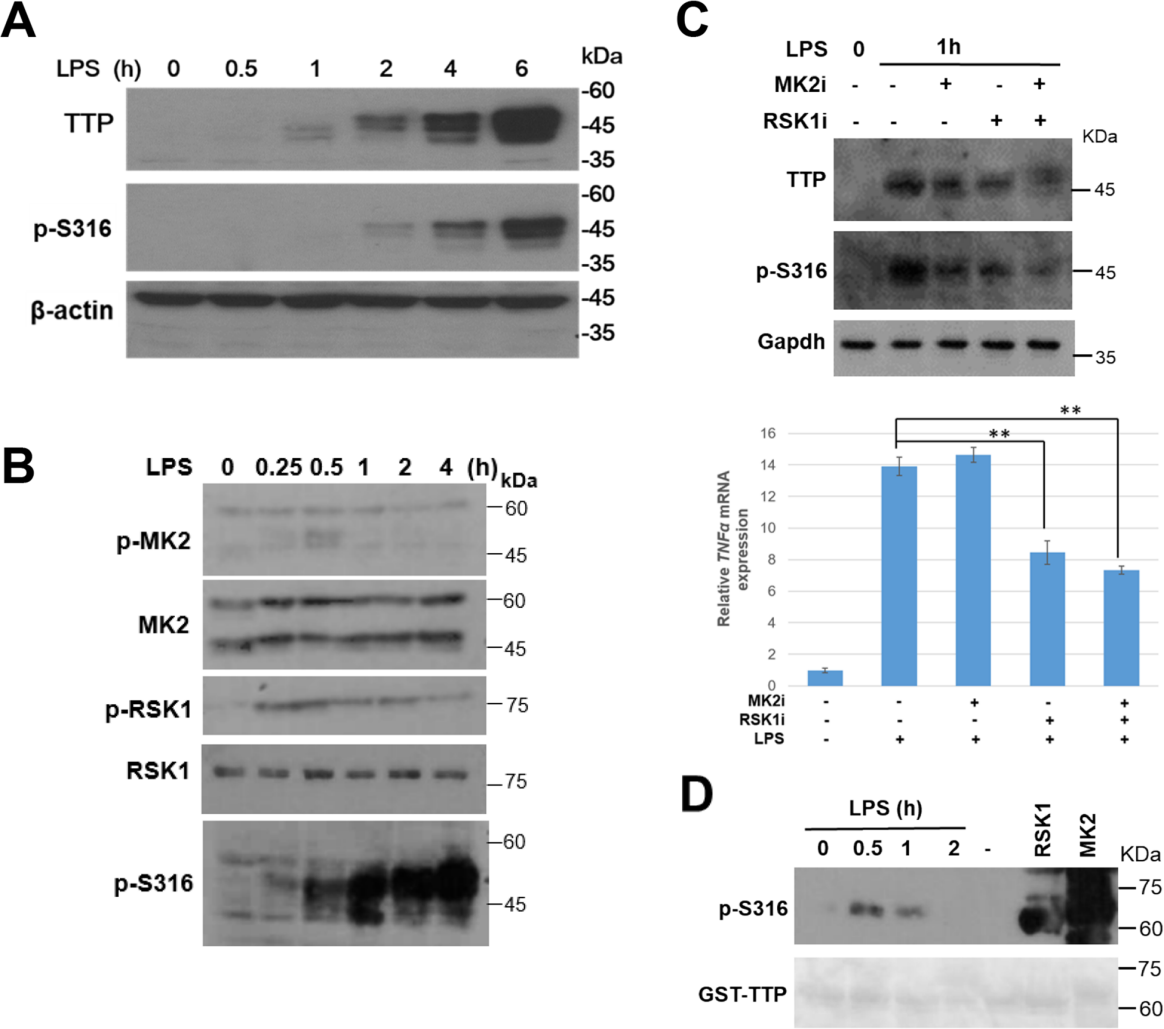
Ser316 is phosphorylated in LPS-treated RAW264.7 cells. **A** The kinetic analysis of Ser316 phosphorylation. RAW264.7 cells were treated with 100 ng/ml of LPS for indicated time intervals. Whole cell extracts were isolated for western blotting analysis as indicated. **B** Whole cell extracts from control and LPS-treated RAW264.7 cells were isolated and western blotting analysis with antibodies as indicated. **C** RAW264.7 cells were pretreated with 2 μM of MK2 (PF3644022) or 25 μM of RSK1(BI-D1870) inhibitors for 0.5 h and followed by LPS stimulation for 1 h. Whole cell extracts were isolated and western blotting analysis with antibodies as indicated, and RNA was isolated for RT-qPCR with control β-actin and TNFα primers. **P<0.01. **D** Solid phase kinase assay. GST-TTP bound on glutathione –sepharose was incubated with or without LPS-treated cell lysates. After extensive washes, kinase assays were performed. RSK1 and MK2 were included as positive controls. The samples were separated on SDS-PAGE and western blotting with anti-p-S316 and then ponceau S staining

To investigate the functional effect of Ser316 phosphorylation, we examine it in LPS-stimulated RAW264.7 macrophages. As shown in Fig. 3 A, LPS induced TTP expression and Ser316 phosphorylation. RSK1 and MK2 also were activated in early LPS stimulation (Fig. 3 B). When RSK1 or MK2 inhibitor was pre-treated for 30 min followed by LPS stimulation for 1 h, Ser316 phosphorylation was slightly decreased, and the typical TTP target mRNA *TNFα* was suppressed in the presence of RSK1 inhibitor (Fig. 3 C). However, the kinase inhibitors treatment showed no significant effect on *TNFα* mRNA stability analysis by adding transcription inhibitor actinomycin D (Fig. S3). The solid phase kinase assays demonstrated that LPS stimulation for 30 min induced Ser316 phosphorylation (Fig. 3 D). Taken together, Ser316 of TTP is phosphorylated in LPS-stimulated RAW264.7 cells, and RSK1 and MK2 might involve this residue’s phosphorylation.

### Generation of TTP knockout RAW264.7 cells using CRISPR/Cas9 genome editing

**Fig. 4:**
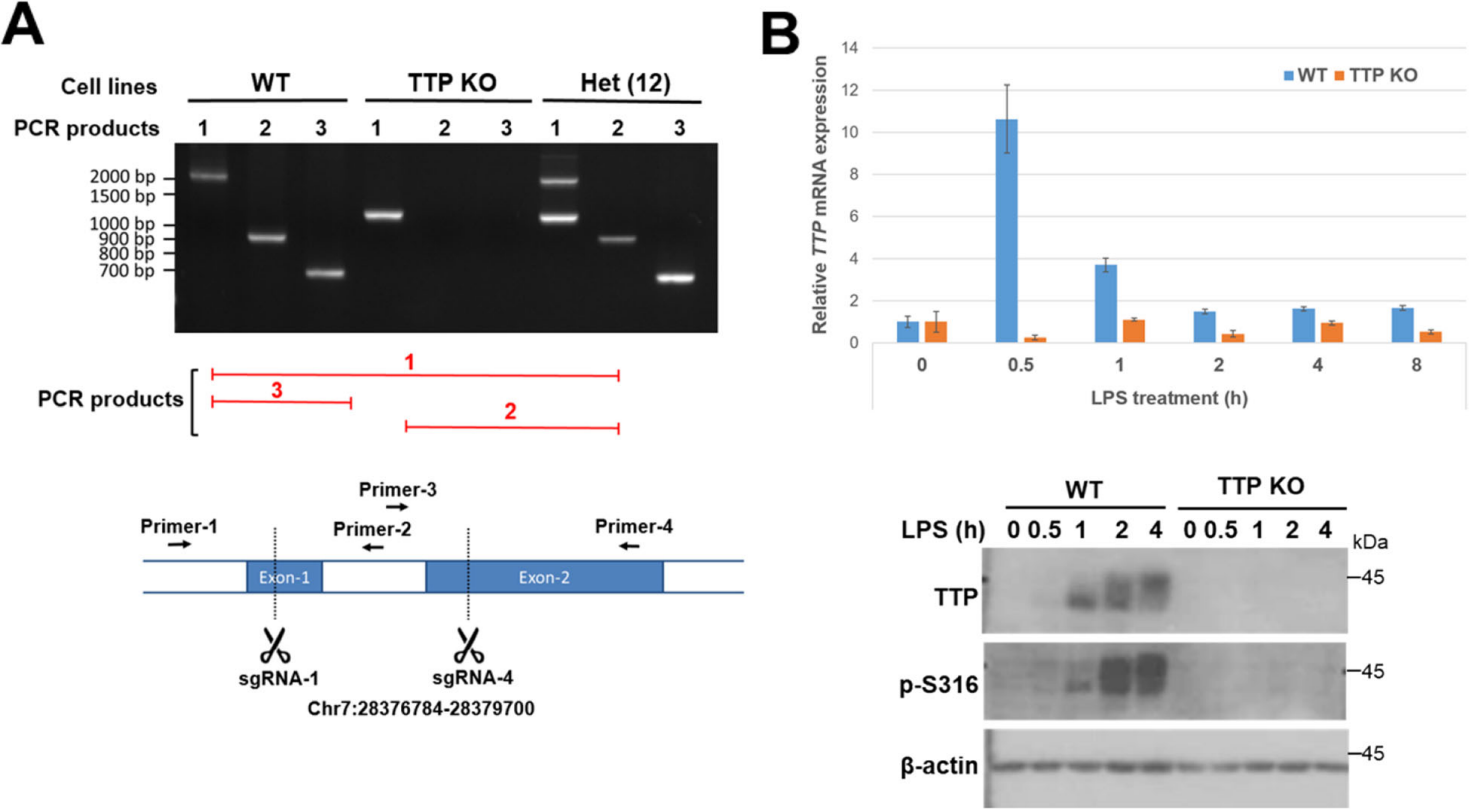
Generation of TTP knockout RAW264.7 cells. **A** The homozygous and heterozygous KO cells were generated by double breaks of CRISPR/Cas9 genome editing in mouse RAW264.7 cells  using sgRNA-1 and sgRNA-4 as indicated. **B** TTP mRNA and protein expression in LPS-stimulated wild-type and TTP KO cells

TTP is highly induced under LPS stimulation to destabilize some cytokines mRNA [41]. We are interested in the functional role of Ser316 phosphorylation on mRNA stabilization. At first, we created TTP knockout (KO) RAW264.7 macrophages using CRISPR/Cas9 genome editing. We induced two double-strand breaks to remove the TTP exon fragment in RAW264.7 genomes (Fig. S4A). Four sgRNAs (single-guide RNAs) (sequences showed in Table S2) were designed by Benchling (https://benchling.com/) to recognize the TTP sequence on mouse genome and were in vitro transcribed (Fig. S4B). Cas9 protein and sgRNAs were prepared and co-transfected into RAW264.7 cells. The combination of sgRNA-1 and sgRNA-4 had the most cutting efficiency which was checked by genomic PCR (Fig. S4C). By flow cytometry and cell sorting, RAW264.7 cells were separated into single-cell for selection. Homozygous and heterozygous TTP KO cell lines were identified by genomic PCR (Fig. S4D). Further check of genomic PCR was designed by using three pairs of primer to demonstrate TTP KO cell lines (Fig. 4 A). The qPCR and western blotting assays were performed to confirm no TTP mRNA and protein expression in KO cells (Fig. 4 B). TTP KO RAW264.7 cells are used to examine the functional effects of phosphorylated mutants.

### The functional effect of Ser316 phosphorylation in LPS-stimulated RAW264.7 cells

**Fig. 5:**
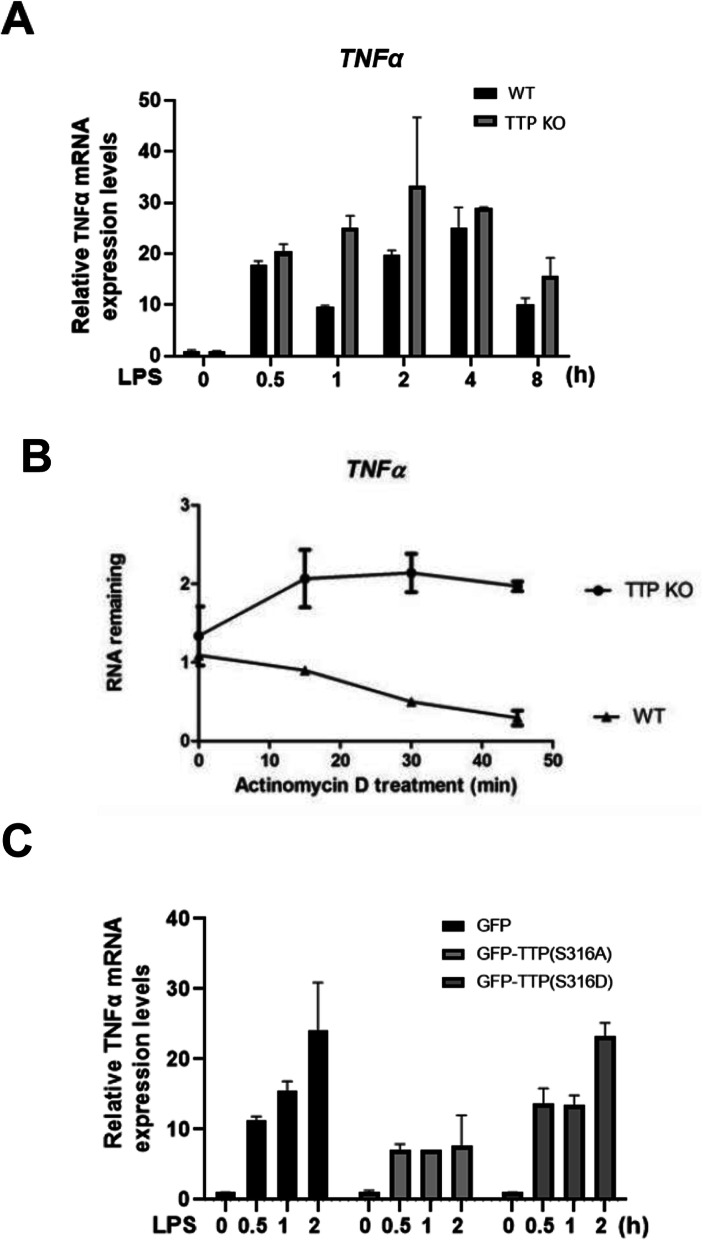
The functional analysis of Ser316 phosphorylation in LPS-stimulated RAW264.7 cells. **A** Kinetics of *TNFα* mRNA expression in LPS-treated wild-type and TTP KO cells. **B** *TNFα* mRNA stability analysis. Wild-type and TTP KO RAW264.7 cells were treated with 100 ng/ml of LPS for 30 min and then adding 10 μg/ml of actinomycin D to block transcription for 15, 30, and 45 min. Total RNAs were isolated to perform RT-qPCR with TNFα and β-actin primers and the percent of RNA remaining was shown. **C** TTP rescue analysis. TTP KO cells were transfected with GFP vector, or GFP-TTP(S316A), or GFP-TTP(S316D). After 24 h, the cells were treated with 100 ng/ml of LPS for 0.5, 1, and 2 h, and RNA was isolated for qPCR analysis with TNFα and β-actin primers. All experiments were performed independently at least three times, and error bars represent mean ± S.D.

The qPCR analysis showed the *TNFα* mRNA expression levels in wild-type and TTP KO cells (Fig. 5 A). Interestingly, *TNFα* mRNA was induced at 30 min and then decreased at 1 h in wild-type cells, while no decrease of *TNFα* at 1 h induction in TTP KO cells. The mRNA stability was analyzed by adding the transcription inhibitor actinomycin D at 30 min (Fig. 5 B). A longer mRNA half-life was observed in TTP KO cells. It indicates that TTP plays a role in the TNFα mRNA decay. To investigate how Ser316 phosphorylation affects *TNFα* mRNA stability, the KO cells were transfected with plasmids expressing GFP-TTP(S316A) or GFP-TTP(S316D) or GFP vector control. After transfection, the cells were treated with LPS to induce *TNFα* expression and RNA was isolated for qPCR analysis (Fig. 5 C). In the presence of TTP (S316A), the *TNFα*’s induction was decreased, indicating the higher suppressive activity of non-phosphorylated mutant S316A than phosphomimetic S316D. The results indicate that Ser316 phosphorylation of TTP decreases its mRNA destabilization activity.

### The molecular mechanism of Ser316 phosphorylation-regulated mRNA expression

**Fig. 6:**
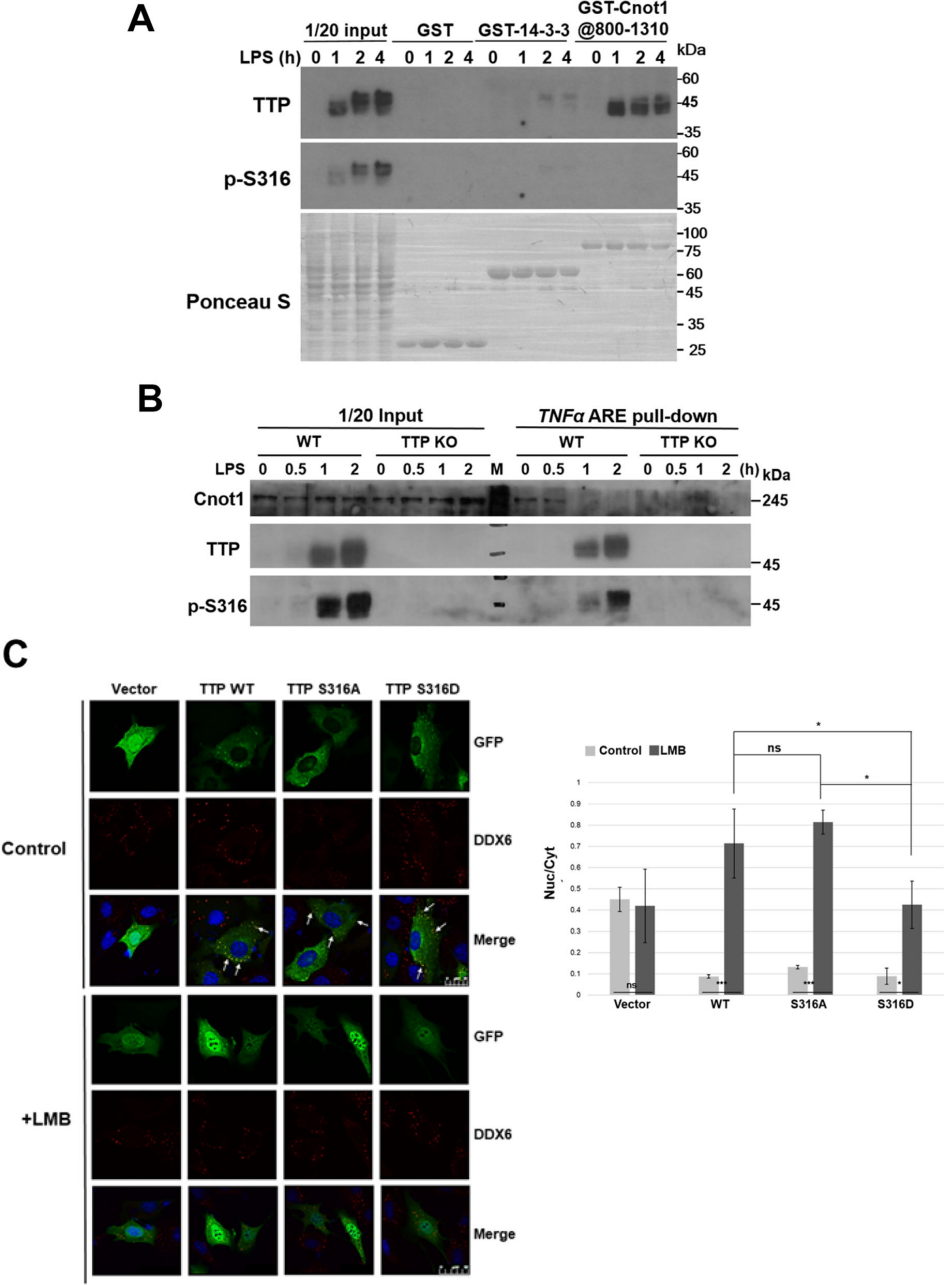
Ser316 phosphorylation impairs TTP interaction with CNOT1 in LPS-stimulated RAW264.7 cells. **A** GST-pull down assay. RAW264.7 cells were treated with 100 ng/ml LPS for 0, 1, 2, and 4 h. Whole cell extracts were harvested and incubated with GSH sepharase-bound GST, or GST-14-3-3, or GST-Cnot1@800–1310. The pull-down protein complexes were analyzed by western blotting with anti-TTP and anti-p-S316 antibodies. **B** RNA pull-down assay. *TNFα* ARE probe was incubated with cell extracts from control and LPS-treated for 0.5 h, 1 h, and 2 h. The pull-down RNA-protein complexes were analyzed by western blotting with anti-TTP, anti-phospho-S316, and anti-Cnot1. **C** Immunofluorescent staining. NIH3T3 cells cultured on coverslips were transfected with GFP, GFP-TTP(WT), GFP-TTP(S316A), or GFP-TTP(S316D) expressing plasmids. After 24 h, the cells were untreated (control) or treated with leptomycin B (LMB) for 5 h. Immunofluorescence was performed using anti-DDX6 as a marker of P-bodies and images were recorded using a confocal microscope. The arrows indicate the association of GFP and DDX6 signals. The size bar in the control panel is 25 μM and in the LMB panel is 50 μM. The right panel was the quantitative immunofluorescence analysis showing the ratio of nuclear and cytoplasm signals. ***P<0.001; *P<0.05; ns: non-significant. At least three independent experiments were performed

To further explore the functional regulation of LPS-stimulated Ser316 phosphorylation, a GST pull-down was performed by using GST-14-3-3 or GST-Cnot1@800–1310 to pull-down LPS-treated RAW264.7 cell extracts (Fig. 6 A). Interestingly, the TTP pulled down by GST-Cnot1 was not recognized by anti-p-Ser316; however, pulled down by GST-14-3-3 exhibited high molecular weight and was detected by anti-p-Ser316. Moreover, we performed an RNA pull-down assay using *TNFα* ARE incubating with LPS-treated cell extracts (Fig. 6 B). TTP with Ser316 phosphorylation can be pulled-down by *TNFα* ARE; however, the Cnot1 was not detected in the pull-down reactions of LPS-stimulated for 1 h and 2 h. These suggest that Ser316 phosphorylation does not alter RNA-binding activity of TTP, but weakens Cnot1 interaction and enhances 14–3-3 interaction. We also performed co-IP and RNA-IP using anti-TTP in the RAW264.7 cytosolic extracts. The results revealed that TTP interacted with Cnot1 in the LPS-treated 0.5 h cells, and TTP-associated *TNFα* mRNA was detected in the LPS-treated 2 h cells (Fig. S5). In addition, TTP is a nucleo-cytoplasmic shuttling protein whose localization is controlled by external stimuli [42]. To further demonstrate the relationship of TTP phosphorylation and the subcellular localization, NIH3T3 cells were overexpressed with GFP-fused proteins for indirect immunofluorescence staining. The previous report had demonstrated that TTP delivered ARE-containing mRNAs to processing-body (P-body) [43]. One component of the mRNA decapping complex, DDX6, is a marker of P-body [44]. As shown in Fig. 6 C, all wild-type, S316A, and S316D mutants were predominantly located in the cytoplasm and showed a similar colocalization with P-body marker DDX6. When cells treated with leptomycin B (LMB) which blocks exportin 1-mediated TTP protein export [45], TTP was majorly in the nucleus, suggesting its nucleocytoplasmic shuttling property. The quantified result exhibited that the ratio of nucleus and cytoplasm in S316D phosphomimetic mutant was lower than wild-type and S316A mutant, indicating phosphorylation at Ser316 slightly inhibited TTP nuclear import (Fig. 6 C). Taken together, TTP might retain in the cytoplasm when Ser316 is phosphorylation.

## Discussion

TTP is a highly phosphorylated protein, and the functional regulation by phosphorylation is an important subject in TTP study [46–48]. We generated a specific antibody against phospho-Ser316 and demonstrated that ERK-RSK1 and p38-MK2 signaling pathways phosphorylate TTP at Ser316 in LPS-stimulated RAW264.7 cells (Figs. 1 and 3). The previous results showing that the TTP-mediated turnover of *TNFα* mRNA is inhibited by the combined activation of ERKs and p38 [33]. Our finding suggests that Ser316 might be involved in this effect through phosphorylated by RSK1 and MK2 in response to differential MAPK signals. The IP, GST pull-down, and RNA pull-down assays (Fig. 2, Fig. 6, and Fig. S5) demonstrated that Ser316 phosphorylation weakens the interaction with CNOT1 in the CCR4-NOT deadenylase complex. It was consistent with a report that described the human TTP peptide (residues 312–326, mouse TTP residues 305–319) containing phospho-S323 (like mouse S316) showed lower CNOT1@800–999 binding affinity than the wild-type peptide [28]. Additionally, the ERK–RSK pathway also phosphorylates one of the TTP family proteins, ZFP36L1, at S334 in the conserved C-terminal NOT1-binding domain and inhibits its interaction with CNOT7 [34]. We also provide evidence to prove the Ser316 phosphorylation was removed by PP2A (Fig. S1B).

CNOT1 is a scaffold protein that interacts with the deadenylase CNOT7 via its central MIF4G domain [49], with CNOT9 via a DUF3819 domain [50, 51], with the CNOT2-CNOT3 heterodimer via a C-terminal SH domain [52, 53], and with CNOT10-CNOT11 via its N-terminus [54]. CNOT6 interacts with CNOT7 via its N-terminal leucine-rich repeat domain, but it does not interact directly with CNOT1 [55]. IP showed that TTP forms a complex with CNOT1, CNOT3, CNOT6, and CNOT7 (Fig. 2 A). When we used tandem mass spectrometry to analyze TTP-associated proteins, several CCR4-NOT complex subunits were detected, including CNOT1, CNOT2, CNOT6, CNOT6L, CNOT10, and CNOT11 (data not shown). Knockdown analyses demonstrated that CNOT1, CNOT6, and CNOT7 play roles in TTP mRNA destabilization activity (Fig. 2 B and Fig. S2). When CNOT1 was knocked down, CNOT6 and CNOT7 could not be co-precipitated with TTP (Fig. 2 B). Therefore, knockdown of CNOT1 had a greater effect on TTP activity than knockdown of CNOT6 or CNOT7 (Fig. S2C). Through the interaction with CNOT1, TTP might communicate with other components of the mRNA decay machinery. The MIF4G domain of CNOT1 interacts with the translation repressor DDX6 to bridge deadenylation and decapping [50, 51, 56]. Recent studies have shown that CNOT9 interacts with GW182/TNRC6C and involves microRNA-mediated repression [50, 51]. However, in our immunofluorescence staining and IP results (Fig. 2 C), the interaction between TTP and DDX6 is phosphorylation independent. TTP might associate with DDX6-containing P-body in RAW264.7 cells (Fig. 6 C) [57].

In response to LPS stimulation, the *TNFα* mRNA was dramatically induced at 30 min and decreased at 1 h (Fig. 5 A), while the decrease was not observed in TTP KO cells, indicating TTP plays a role in this response. When ectopic expression of TTP S316A or TTP S316D in TTO KO cells, the decrease at 1 h was not recovered (Fig. 5 C). We suggest that the dynamic TTP phosphorylation is required for the bi-phasic *TNFα* expression [17, 58], and the lower amount and hypo-phosphorylated TTP at 1 h induction (Fig. 4 A) exhibits higher mRNA destabilization activity. Like phosphorylation at serines 52 and 178 by p38-MK2 signaling [32], Ser316 phosphorylation of TTP also displayed cytoplasmic localization (Fig. 6 C). It might be due to the interaction between hyper-phosphorylated TTP and 14–3-3 protein (Fig. 6 A). We observed serines 52 /178 and Ser316 played functions independently in the recruitment of the CCR4-NOT deadenylase complex. That is TTP phosphorylation on either Ser52/178 or Ser316 would decrease association with CCR4-NOT complex (Fig. 2 D). It is consistent with a recent report in knockout mice study showing that Ser316 is another residue phosphorylated by MK2/3 in addition to serines 52 and 178, and they regulate TNF biosynthesis independently [35]. However, the Ser316 phosphorylation seems weaker in TTP Ser52,178A mutant than wild-type (Fig. 1 A and D). Whether phosphorylation at serines 52 and 178 affects Ser316 phosphorylation will be further clarified and investigated. TTP contains intrinsically disordered regions (IDRs) which facilitate rapid degradation of TTP protein [15]. The serines 52 and 178 are located in IDR, and those phosphorylations can inhibit TTP protein degradation [32, 58]. Ross and his colleagues generated the mouse strain expressed TTP-S52,178A, and the mutant protein was unstable and expressed low levels in mice, but it functioned higher mRNA destabilization activity than wild-type [27]. TTP-S316A or -S316D mutants did not alter their protein half-lives in our preliminary examination. Our results imply the complex regulation of TTP phosphorylation, which might control a network of protein-protein interaction to modulate target mRNA stability.

## Conclusions

**Fig. 7:**
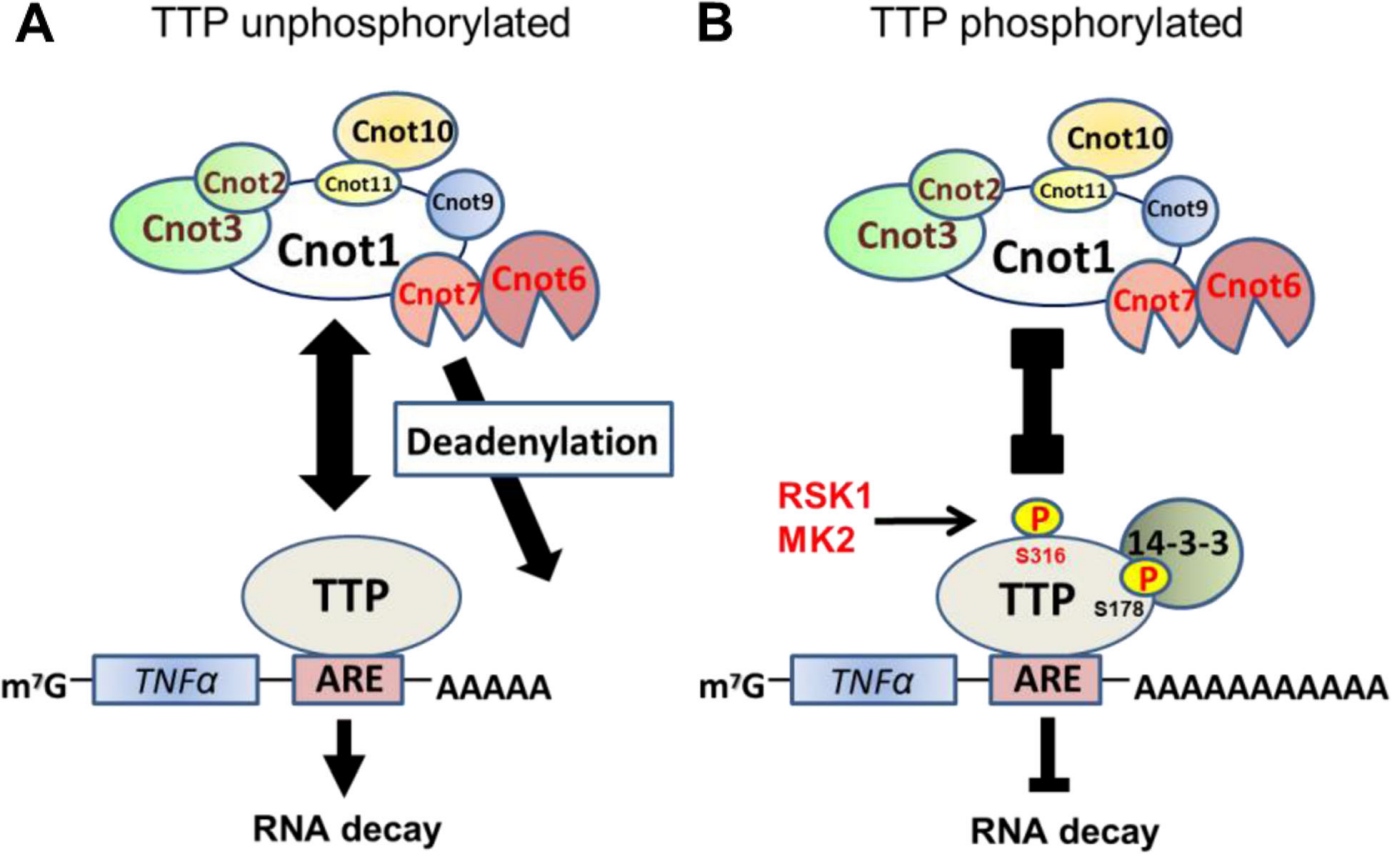
The TTP-CCR4-NOT-mRNA decay axis is regulated by Ser316 phosphorylation. **A** TTP interacts with CCR4-NOT deadenylase complex via CNOT1 under Ser316 unphosphorylated status. CNOT6 and CNTO7 would deadenylate TTP target ARE-containing mRNA such as *TNFα* and lead to mRNA decay. **B** RSK1 and MK2 are activated by inflammatory stimuli to phosphorylate Ser316, attenuating the interaction between TTP and CCR4-NOT complex. Ser178 is also phosphorylated to facilitate TTP and 14–3-3 interaction and independently inhibits the TTP-CCR4-NOT-mRNA decay axis

The inflammatory stimulus such as LPS activates ERK-RSK1 and p38-MK2 signaling, resulting in phosphorylation at Ser316 in the C-terminal NOT1-binding domain of TTP. TTP and CCR4-NOT deadenylase complex interaction is impaired by this phosphorylation, leading to TTP target mRNAs such as *TNFα* stabilized (Fig. 7).

## Supplementary Information

**Additional file 1: Figure S1.** (A) ERK and p38 signaling pathways result in S316 phosphorylation. Two hundred ninety three T cells were transfected with constitutive-active (CA) or dominant-negative (DN) MKK1 or MKK3 expression plasmids. After treated with RSK1 inhibitor (RSKi: 50 μM of BD-I1870) or MK2 inhibitor (MK2i: 5 μM of PF364402) for 2 h, the whole cell extracts were isolated for western blotting analysis with indicating antibodies. (B) PP2A decreases Ser316 phosphorylation. HEK293T cells were coexpressed with wild type or S316A or S52, 178A TTP, and the wild type or non-activity mutant H59Q of the catalytic subunit of PP2Ac or the regulatory subunit PR55. The western blotting was performed with indicated antibodies. **Figure S2.** (A) S316 is critical for TTP binding to CNOT1. HEK293T cells were transfected with 4 μg of Flag-tagged TTP expression plasmids as indicated. IP was performed with anti-Flag M2 agarose, and the precipitated protein complexes were analyzed by western blotting with indicated antibodies (B) Luciferase reporter assay of TTP phosphomimetic mutants. Schematic diagram of MKP-1-3’UTR (three AREs) containing luciferase reporter. The TTP S316D mutant resulted in higher luciferase activity than wild-type. * *P* < 0.05. (C) Knockdown of CCR4-NOT complex reduces TTP mRNA-destabilizing ability. HEK293T cells were seeded in 12-well culture plates and transfected with 5 nM of siRNA targeting CNOT1, CNOT6, CNOT7, and negative control (NC). After 24 h, cells were transfected again with 0.2 μg of Flag-tagged TTP, 0.5 μg of luciferase reporter carrying MKP-1-3’UTR or reporter alone, and 0.5 μg of Renilla luciferase reporter (served as an internal control). Dual-luciferase reporter assays were performed after 24 h post-transfection. The relative MKP-1-3’UTR-mediated luciferase activities were normalized to the Renilla luciferase activities and to that of the reporter alone. Each treatment group contained two duplicates, and experiments were repeated three times. Data are presented as means ± SD. The protein expression level and knockdown efficiency were examined by western blot analysis (lower panel) using indicated antibodies. The asterisk indicates Flag-tagged firefly luciferase. (D) Another IP result of Fig. 2 C. **Figure S3** *TNFα* mRNA stability analysis. RAW264.7 cells were pre-treated with RSK1 inhibitor (RSKi: 50 μM of BD-I1870) or together with MK2 inhibitor (MK2i: 5 μM of PF364402) for 30 min, and then treated with 100 ng/ml of LPS for 1 h. The cells were added transcription inhibitor actinomycin D (Act.D, 10 μg/ml) for 15 min, 30 min, and 45 min. The cells were harvested for RNA isolation and RT-qPCR analysis. **Figure S4.** The generation of TTP KO RAW264.7 cells. (A) The genomic *TTP* sequence is located on chromatin7:28376784–28,379,700 which has two exons and one intron. The four sgRNAs were designed to recognize the specific unique sequence positioned on *TTP* exons that contain NGG, which were assembled with T7 promoter and generated by in vitro transcription. (B) Different combinations of sgRNAs were co-transfected with Cas9 protein in RAW264.7 cells and checked by genomic PCR (Primers showed in Table S2). The genomic knock-out PCR products were predicted as red arrows. (C) Fifteen cell lines of RAW264.7 cells were checked by genomic PCR. The number 9 was a possible homozygous KO cell, and number 12 is one of the heterozygous clones. **Figure S5.** Co-immunoprecipitation and RNA-immunoprecipitation (IP) in LPS-stimulated RAW264.7 cells with anti-TTP. To prepare cytosolic extract, 5 × 10^6^ cells were resuspended in 400 μl of hypotonic buffer (10 mM HEPES pH 7.5, 10 mM KCl, 1.5 mM MgCl_2_, 2.5 mM DTT, 0.05% NP-40 with protease and phosphatase inhibitors). The cell suspension was on ice for 15 min, and then 25 μl of 10% NP-40 was added followed by vortexing for 10 s. After centrifugation at 10,000×g for 30 s, the supernatant was collected as cytoplasmic extract. 1 mg cytoplasmic extracts from RAW264.7 cells were adjusted to 25 mM HEPES, pH 7.5, 150 mM 5NaCl, 1.5 mM MgCl_2_, 0.2 mM EDTA, 0.1% Triton X-100, 0.5 mM DTT and 1u/μl of RNasin and were pre-cleaned by protein-A Sepharose (Amershan Pharmacia) for 1 h. After centrifugation, the supernatants were added 1 μg of normal IgG or anti-TTP antibody and protein A-Sepharose at 4 °C rotated for 2 h. Beads were washed using NT2 buffer (50 mM Tris-HCl, pH 7.4, 150 mM NaCl, 1 mM MgCl_2_, and 0.05% NP-40) for three times. For co-IP, the precipitated protein complexes were added with SDS-PAGE sample buffer, boiling for 10 min, and analyzed by western blotting with anti-Cnot1 and anti-TTP (A). For RNA-IP, the beads were incubated with 100 μl NT2 buffer containing 5 U RNase-free DNase I (Ambion) for 15 min at 30 °C, washed with NT2 buffer, and further incubated in 100 μl NT2 buffer containing 0.1% SDS and 0.5 mg/ml proteinase K at 55 °C for 15 min. RNA was extracted with TRIzol reagent and reverse transcribed in cDNAs as mentioned above for semi-quantitative PCR analysis. The specific primers of Gapdh and TNFα was amplified using 5% of the cDNAs from IP and 2% from input in 20 μl containing 10 pmol of forward and reverse primer as shown in Table S1, and lypholized Taq DNA polymerase, buffer and dNTPs (LTK, Inc. Taiwan). PCR was performed in a Robocycler gradient 96 PCR thermal machine (Stratagene) using the following conditions: 95 °C (3 min) for one cycle, 95 °C (30 s), 55 °C (30 s), 72 °C (20 s) for 35 cycles, and a final incubation at 72 °C for 3 min. One-third of PCR products were separated in 2% agarose gel (B). **Table S1.** Primers for generating murine TTP mutants. **Table S2.** Sequences for TTP knock-out in CRISPR/Cas9 system.

## Data Availability

The data and materials that supporting the findings of this study are available on request from the corresponding author [CJC].

## Abbreviations

ARE: AU-rich element
Caf1: Ccr4-associated factor 1
Ccr4: Carbon catabolite repressor protein 4
CRISPR/Cas9: Clustered regularly interspaced short palindromic repeats/CRISPR-associated protein nuclease
MAPK: Mitogen-activated protein kinase
ERK: Extracellular signal-regulated kinase
GST: Glutathione S-transferase
IL: Interleukin
IP: Immunoprecipitation
LPS: Lipopolysaccharide
MK2: MAPK p38-activated kinase 2
NOT1: Negative on TATA 1
PP2A: Protein phosphatase 2A
RSK1: p90 ribosomal S6 kinase 1
sgRNA: Single-guide RNA
TNF: Tumor necrosis factor
TTP: Tristetraprolin
UTR: Untranslated region
Zfp36l1: Zinc finger protein 36, C3H type-like 1
