# Supplementary material for: The functional characterization of phosphorylation of tristetraprolin at C-terminal NOT1-binding domain
Source: J Inflamm (Lond). 2021 Jun 5;18:22. doi: 10.1186/s12950-021-00288-2 (PMC8180021; doi:10.1186/s12950-021-00288-2)
Supplement: Supplementary file 1 — Additional file 1: Figure S1. (A) ERK and p38 signaling pathways result in S316 phosphorylation. Two hundred ninety three T cells were transfected with constitutive-active (CA) or dominant-negative (DN) MKK1 or MKK3 expression plasmids. After treated with RSK1 inhibitor (RSKi: 50 μM of BD-I1870) or MK2 inhibitor (MK2i: 5 μM of PF364402) for 2 h, the whole cell extracts were isolated for western blotting analysis with indicating antibodies. (B) PP2A decreases Ser316 phosphorylation. HEK293T cells were coexpressed with wild type or S316A or S52, 178A TTP, and the wild type or non-activity mutant H59Q of the catalytic subunit of PP2Ac or the regulatory subunit PR55. The western blotting was performed with indicated antibodies. Figure S2. (A) S316 is critical for TTP binding to CNOT1. HEK293T cells were transfected with 4 μg of Flag-tagged TTP expression plasmids as indicated. IP was performed with anti-Flag M2 agarose, and the precipitated protein complexes were analyzed by western blotting with indicated antibodies (B) Luciferase reporter assay of TTP phosphomimetic mutants. Schematic diagram of MKP-1-3’UTR (three AREs) containing luciferase reporter. The TTP S316D mutant resulted in higher luciferase activity than wild-type. * P < 0.05. (C) Knockdown of CCR4-NOT complex reduces TTP mRNA-destabilizing ability. HEK293T cells were seeded in 12-well culture plates and transfected with 5 nM of siRNA targeting CNOT1, CNOT6, CNOT7, and negative control (NC). After 24 h, cells were transfected again with 0.2 μg of Flag-tagged TTP, 0.5 μg of luciferase reporter carrying MKP-1-3’UTR or reporter alone, and 0.5 μg of Renilla luciferase reporter (served as an internal control). Dual-luciferase reporter assays were performed after 24 h post-transfection. The relative MKP-1-3’UTR-mediated luciferase activities were normalized to the Renilla luciferase activities and to that of the reporter alone. Each treatment group contained two duplicates, and experiments were repeated three tim [file 12950_2021_288_MOESM1_ESM.docx]

**Figure S1**


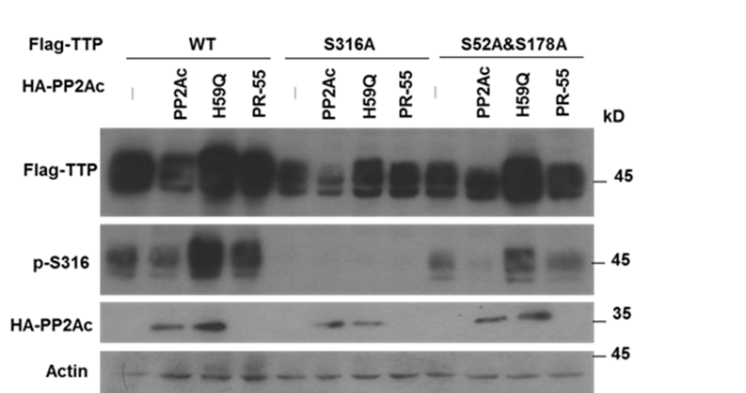

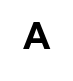

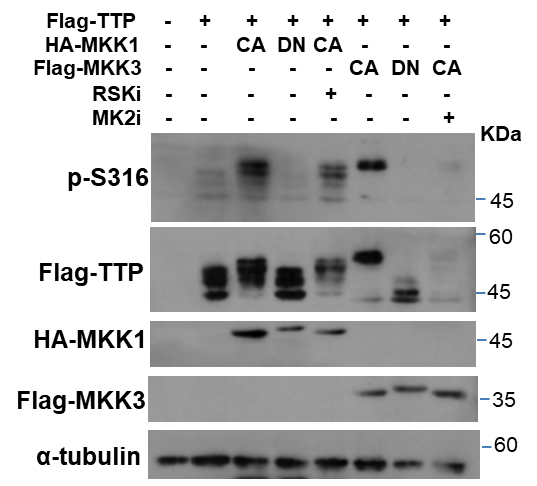

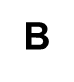


**Fig.S1** (A) ERK and p38 signaling pathways result in S316 phosphorylation. 293T cells were transfected with constitutive-active (CA) or dominant-negative (DN) MKK1 or MKK3 expression plasmids. After treated with RSK1 inhibitor (RSKi: 50 μM of BD-I1870) or MK2 inhibitor (MK2i: 5 μM of PF364402) for 2 h, the whole cell extracts were isolated for western blotting analysis with indicating antibodies. (B) PP2A decreases Ser316 phosphorylation. 293T cells were coexpressed with wild type or S316A or S52, 178A TTP, and the wild type or non-activity mutant H59Q of the catalytic subunit of PP2Ac or the regulatory subunit PR55. The western blotting was performed with indicated antibodies


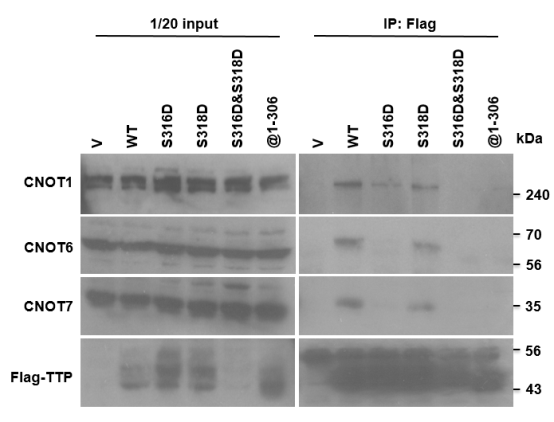

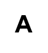
**
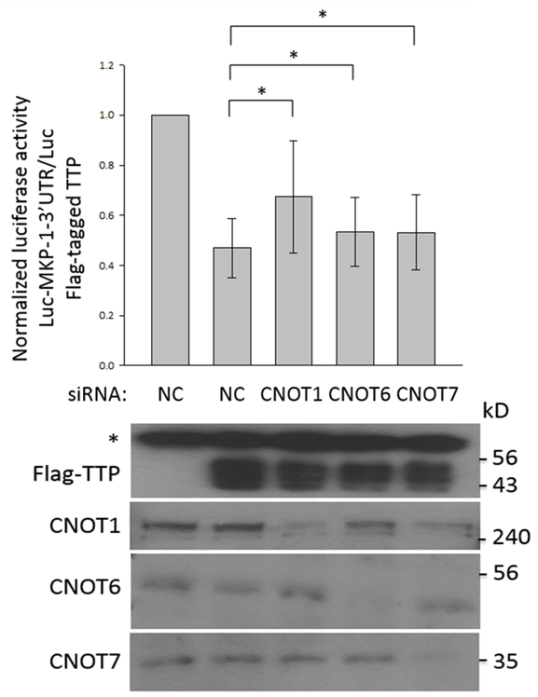
**
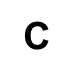
 **Figure S2**


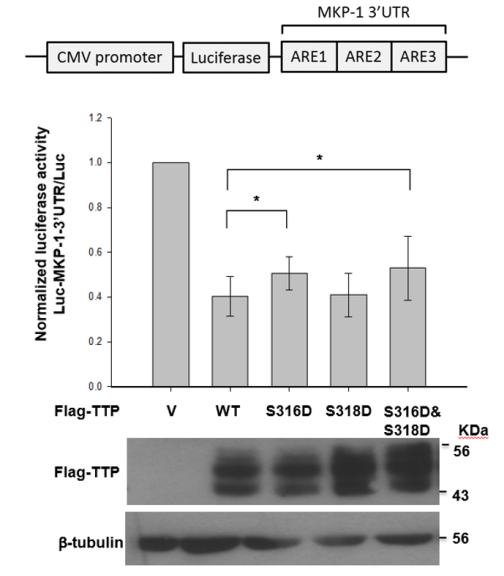
**
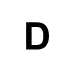
**
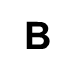


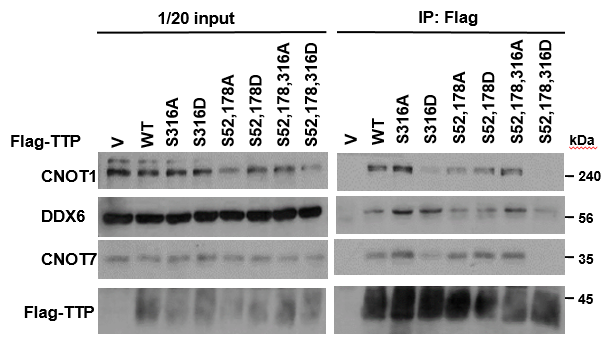


**Fig.S2** (A) S316 is critical for TTP binding to CNOT1. 293T cells were transfected with 4 μg of Flag-tagged TTP expression plasmids as indicated. IP was performed with anti-Flag M2 agarose, and the precipitated protein complexes were analyzed by western blotting with indicated antibodies (B) Luciferase reporter assay of TTP phosphomimetic mutants. Schematic diagram of MKP-1-3’UTR (three AREs) containing luciferase reporter. The TTP S316D mutant resulted in higher luciferase activity than wild-type. * p<0.05. (C) Knockdown of CCR4-NOT complex reduces TTP mRNA-destabilizing ability. 293T cells were seeded in 12-well culture plates and transfected 5 nM of siRNA targeting CNOT1, CNOT6, CNOT7, and negative control (NC). After 24 hours, cells were transfected again with 0.2 μg of Flag-tagged TTP, 0.5 μg of luciferase reporter carrying MKP-1-3’UTR or reporter alone, and 0.5 μg of Renilla luciferase reporter (served as an internal control). Dual-luciferase reporter assays were performed after 24 h post-transfection. The relative MKP-1-3’UTR-mediated luciferase activities were normalized to the Renilla luciferase activities and to that of the reporter alone. Each treatment group contained two duplicates, and experiments were repeated three times. Data are presented as means ± SD. The protein expression level and knockdown efficiency were examined by western blot analysis (lower panel) using indicated antibodies. The asterisk indicates Flag-tagged firefly luciferase. (D) Another IP result of Fig.2C.

**Figure S3**

**
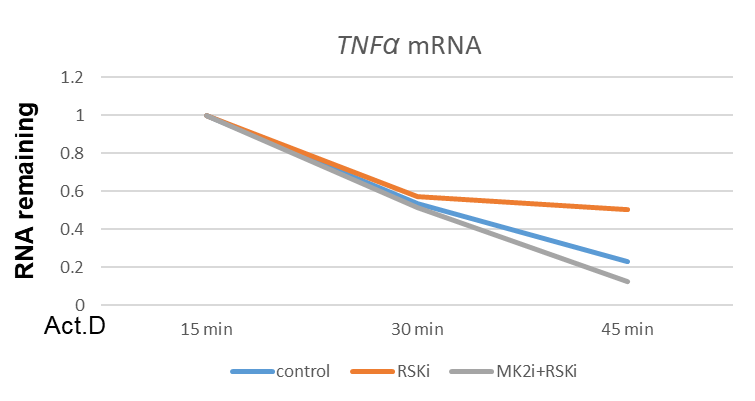
**

**Fig.S3** TNFα mRNA stability analysis. RAW264.7 cells were pre-treated with RSK1 inhibitor (RSKi: 50 μM of BD-I1870) or together with MK2 inhibitor (MK2i: 5 μM of PF364402) for 30 min, and then treated with 100 ng/ml of LPS for 1 h. The cells were added transcription inhibitor actinomycin D (Act.D, 10 μg/ml) for 15 min, 30 min, and 45 min. The cells were harvested for RNA isolation and RT-qPCR analysis.

**Figure S4**

**
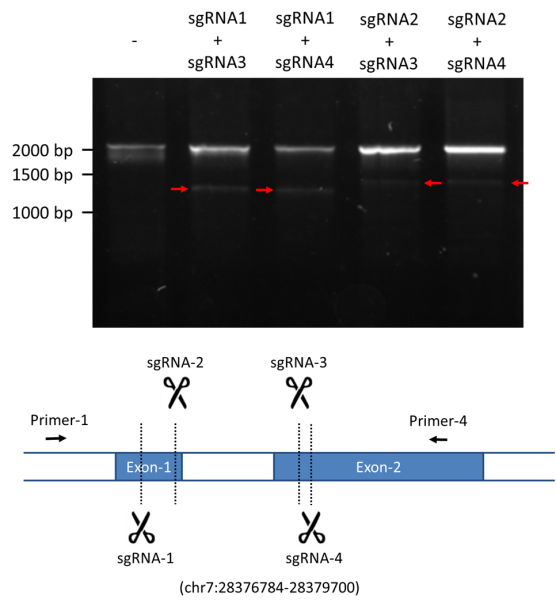
**

**BB**

**A**

**
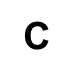
**

**
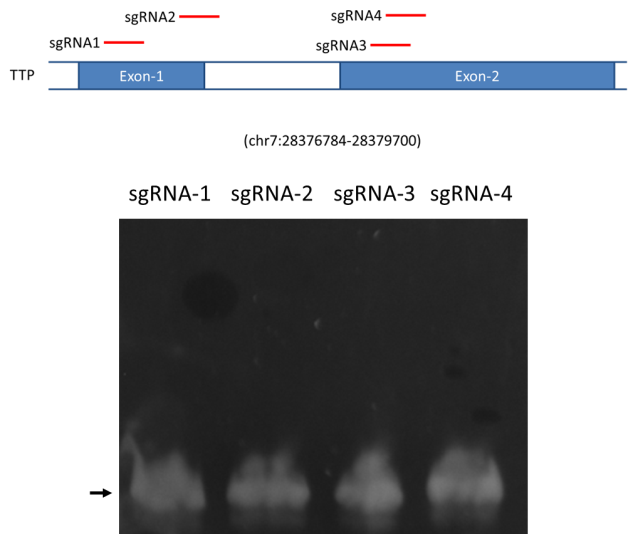

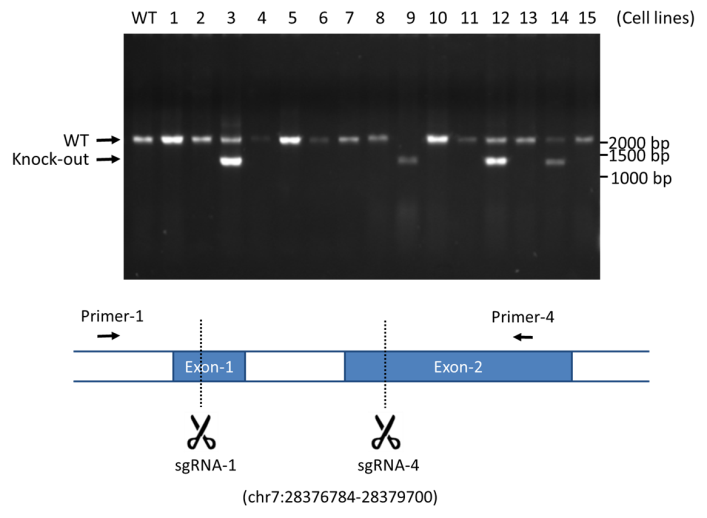
Fig.S4** The generation of TTP KO RAW264.7 cells. (A) The genomic TTP sequence is located on chromatin7:28376784-28379700 which has two exons and one intron. The four sgRNAs were designed to recognize the specific unique sequence positioned on TTP exons that contain NGG, which were assembled with T7 promoter and generated by in vitro transcription. (B) Different combinations of sgRNAs were co-transfected with Cas9 protein in RAW264.7 cells and checked by genomic PCR (Primers showed in Table S2). The genomic knock-out PCR products were predicted as red arrows. (C) Fifteen cell lines of RAW264.7 cells were checked by genomic PCR. The number 9 was a possible homozygous KO cell, and number 12 is one of the heterozygous clones.

**C**

**Figure S5**


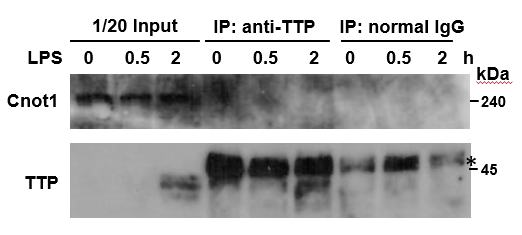

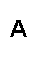


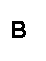

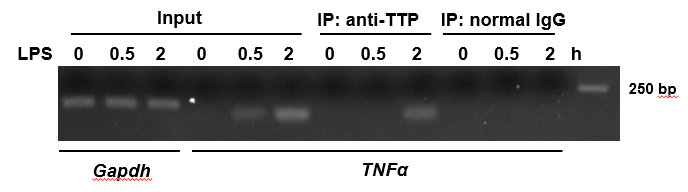


Fig.S5 Co-immunoprecipitation and RNA-immunoprecipitation (IP) in LPS-stimulated RAW264.7 cells with anti-TTP. To prepare cytosolic extract, 5x10^6^ cells were resuspended in 400 μl of hypotonic buffer (10 mM HEPES pH7.5, 10 mM KCl, 1.5 mM MgCl_2_, 2.5 mM DTT, 0.05% NP-40 with protease and phosphatase inhibitors). The cell suspension was on ice for 15 min, and then 25 μl of 10 % NP-40 was added followed by vortexing for 10 seconds. After centrifugation at 10,000xg for 30 seconds, the supernatant was collected as cytoplasmic extract. 1 mg cytoplasmic extracts from RAW264.7 cells were adjusted to 25 mM HEPES, pH 7.5, 150 mM 5NaCl, 1.5 mM MgCl_2_, 0.2 mM EDTA, 0.1 % Triton X-100, 0.5 mM DTT and 1u/μl of RNasin and were pre-cleaned by protein-A Sepharose (Amershan Pharmacia) for 1 hr. After centrifugation, the supernatants were added 1 μg of normal IgG or anti-TTP antibody and protein A-Sepharose at 4^o^C rotated for 2 hr. Beads were washed using NT2 buffer (50 mM Tris-HCl, pH 7.4, 150 mM NaCl, 1 mM MgCl_2_, and 0.05 % NP-40) for three times. For co-IP, the precipitated protein complexes were added with SDS-PAGE sample buffer, boiling for 10 min, and analyzed by western blotting with anti-Cnot1 and anti-TTP (A). For RNA-IP, the beads were incubated with 100 μl NT2 buffer containing 5U RNase-free DNase I (Ambion) for 15 min at 30^o^C, washed with NT2 buffer, and further incubated in 100 μl NT2 buffer containing 0.1 % SDS and 0.5 mg/ml proteinase K at 55^o^C for 15 min. RNA was extracted with TRIzol reagent and reverse transcribed in cDNAs as mentioned above for semi-quantitative PCR analysis.　The specific primers of *Gapdh* and *TNFα* was amplified using 5 % of the cDNAs from IP and 2% from input in 20 µl containing 10 pmol of forward and reverse primer as shown in Table1, and lypholized Taq DNA polymerase, buffer and dNTPs (LTK, Inc. Taiwan). PCR was performed in a Robocycler gradient 96 PCR thermal machine (Stratagene) using the following conditions: 95°C (3 min) for one cycle, 95°C (30 sec), 55°C (30 sec), 72°C (20 sec) for 35 cycles, and a final incubation at 72°C for 3 min. One-third of PCR products were separated in 2% agarose gel (B).

| **Table S1. Primers for generating murine TTP mutants** | | |
| --- | --- | --- |
|  | **Forward Primer (5'－﹥3')** | **Reverse Primer (5'－﹥3')** |
| S52A | GCCGCTCCACTGCCCTGGTGGAGGG | CAGTCAGGCGAGAGGTGACCC |
| S52D | GCCGCTCCACTGACCTGGTGGAGGG | CAGTCAGGCGAGAGGTGACCC |
| S178A | GACAAAGCATCGCCTTCTCCGGCTTG | GCAGCACATGGGGCTGGCCAG |
| S178D | GACAAAGCATCGACTTCTCCGGCTTG | GCAGCACATGGGGCTGGCCAG |
| S316A | TCACTCAGAGACAGCGATACGATTG | ATGGATCTCGCCATCTAC |
| S316D | TCACTCAGAGACATCGATACGATTG | ATGGATCTCGCCATCTAC |
| S318A | TCACTCAGCGACAGAGATACGATTG | ATGGATCTCGCCATCTAC |
| S318D | TCACTCATCGACAGAGATACGATTG | ATGGATCTCGCCATCTAC |
| S316A&S318A | TCACTCAGCGACAGCGATACGATTG | ATGGATCTCGCCATCTAC |
| S316D&S318D | TCACTCATCGACATCGATACGATTG | ATGGATCTCGCCATCTAC |

**Table S2. Sequences for TTP knock-out in CRISPR/Cas9 system**

| TTP knock-out | sgRNA-1 | 5’-AATACGACTCACTATAGTTGGTGAAGAGA CCGACTGTGTTTTAGAGCTATGCTGGAAAC AGCATAGCAAGTTAAA-3’ |
| --- | --- | --- |
|  | sgRNA-2 | 5’-TAATACGACTCACTATAGGCCTAAGTTTG GTGCGTCGGTTTTAGAGCTATGCTGGAAAC AGCATAGCAAGTTAAA-3’ |
|  | sgRNA-3 | 5’-TAATACGACTCACTATAGACCTGTCATCC GACCACGGGTTTTAGAGCTATGCTGGAAAC AGCATAGCAAGTTAAA-3’ |
|  | sgRNA-4 | 5’-TAATACGACTCACTATAGGAGGAACCGAA TCCCTCGGGTTTTAGAGCTATGCTGGAAAC AGCATAGCAAGTTAAA-3’ |
| sgRNA scaffold (bottom) | | 5’-GCACCGACTCGGTGCCACTTTTTCAAGTT GATAACGGACTAGCCTTATTTTAACTTGCTAT GCTGTTTCCAGCAT-3’ |
| sgRNA assemble | T7 oligo | 5’-TAATACGACTCACTATAG-3’ |
|  | sgRNA-reverse | 5’-GCACCGACTCGGTGCCACTTTTTCAAG-3’ |
| Genomic PCR check | Primer-1 (F) | 5’-CTTCCACCTCTGAAAAACACTAGGAC-3’ |
|  | Primer-2 (R) | 5’-ATCTCTCTAGAACTCTTACCCTGTCA-3’ |
|  | Primer-3 (F) | 5’-TTACCTTTTCTGGTGGCCTAACCGA-3’ |
|  | Primer-4 (R) | 5’-TCTTTGAGATCTAGCTGATCCATACTG-3’ |
